# The Prognostic Role of Hematological Markers in Acute Pulmonary Embolism: Enhancing Risk Stratification

**DOI:** 10.3390/medicina61061095

**Published:** 2025-06-17

**Authors:** Elena Emilia Babes, Andrei-Flavius Radu, Victor Vlad Babeş, Paula Ioana Tunduc, Ada Radu, Gabriela Bungau, Cristiana Bustea

**Affiliations:** 1 Doctoral School of Biomedical Sciences, University of Oradea, 410087 Oradea, Romania; eebabes@uoradea.ro (E.E.B.); blaj.ioanapaula@student.uoradea.ro (P.I.T.); adaradu@uoradea.ro (A.R.); gbungau@uoradea.ro (G.B.); cbustea@uoradea.ro (C.B.); 2 Department of Medical Disciplines, Faculty of Medicine and Pharmacy, University of Oradea, 410073 Oradea, Romania; 3 Department of Psycho-Neurosciences and Recovery, Faculty of Medicine and Pharmacy, University of Oradea, 410073 Oradea, Romania; 4 Department of Pharmacy, Faculty of Medicine and Pharmacy, University of Oradea, 410028 Oradea, Romania; 5 Department of Preclinical Disciplines, Faculty of Medicine and Pharmacy, University of Oradea, 410073 Oradea, Romania

**Keywords:** pulmonary embolism, risk stratification, hematologic parameters, neutrophil-to-lymphocyte ratio

## Abstract

*Background and Objectives*: Assessing risk is essential for optimal care in acute pulmonary embolism (PE). The present research seeks to evaluate the value of admission blood cellular indices as predictors of in-hospital outcome in acute PE and their utility in conjunction with validated risk tools such as the Pulmonary Embolism Severity Index (PESI) score and the European Society of Cardiology (ESC) risk stratification. *Materials and Methods*: A total of 1058 individuals hospitalized at Bihor County Emergency Hospital, Oradea, Romania, with a diagnosis of acute PE confirmed by contrast-enhanced computed tomographic pulmonary angiography were retrospectively evaluated. *Results*: A total of 165 patients (18.2%) experienced adverse outcomes, including in-hospital mortality, cardiac arrest, cardiogenic shock, or persistent hypotension, and required rescue thrombolytic therapy. The neutrophil-to-lymphocyte ratio (NLR) was an independent predictor for in-hospital adverse outcome OR = 1.071 (95% CI 1.01–1.137), *p* < 0.001. NLR as a predictor of adverse outcome had an AUC of 0.712 (95% CI 0.661–0.742), *p* < 0.001, sensitivity of 72.56%, and specificity of 64.19% for a cutoff value of >5.493. In a combined model with PESI or with ESC risk classification, NLR is leading to a significant improvement in their AUC (*p* < 0.001). *Conclusions*: Among hematological markers, NLR holds the greatest relevance for stratifying risk in acute pulmonary embolism and serves as an independent indicator of unfavorable in-hospital prognosis. NLR had an acceptable discriminative power to predict short-term complications and can increase the predictive value of the PESI score and of ESC risk classification.

## 1. Introduction

Following myocardial infarction and cerebrovascular accidents, acute pulmonary embolism (PE) is the third most common cause of cardiovascular death [1]. Patients with PE exhibit a diverse spectrum of clinical manifestations and varying degrees of severity. Short-term mortality is variable, from 2% in low-risk patients up to 95% in patients at high risk experiencing cardiac arrest.

Risk stratification and prognostic evaluation are crucial for better management of patients with acute PE. Clinical and imagistic parameters and different biomarkers have been studied as prognostic factors of short-term outcome in acute PE [2]. Risk assessment based on clinical tools such as the Pulmonary Embolism Severity Index (PESI) and its simplified version (sPESI) performs better to identify low-risk patients [3]. The accuracy of the PESI index is suboptimal for identifying high risk [2]. Risk stratification in pulmonary embolism, as outlined by the European Society of Cardiology (ESC) guidelines, plays a critical role in assessing the risk of early mortality (either during hospitalization or within 30 days) in affected individuals. This evaluation is crucial for determining the most suitable therapeutic approach. High- and intermediate-high-risk patients often necessitate intensive care observation, potential reperfusion interventions, and hemodynamic stabilization, while low-risk cases may be safely managed at home [2].

Venous thromboembolism (VTE) is associated with activation of inflammatory and coagulation cascades [4]. The inflammatory and coagulation imbalances may be reflected in hematologic abnormalities that can be detected by studying different blood cellular indices. There is growing evidence regarding the value of various hematological parameters obtained from routinely determined complete blood count tests in risk stratification of patients with VTE [5]. Different parameters, such as leukocytes and neutrophils [6], mean platelet volume (MPV) [7,8], red cell distribution width (RDW) [9], anemia [10], and blood cellular ratios, were studied as predictors of all-cause mortality. Adding different blood cellular indices to existing risk stratification models may further improve the accuracy of currently used algorithms [11]. The value of RDW for risk stratification and as a predictor of all-cause mortality in PE was revealed in a few previous studies [11,12,13,14], and furthermore, RDW improved the accuracy of the composite sPESI model in the study by Slajus B. et al. [11]. At the same time, anisocytosis is not specific and has an unclear pathophysiology in PE, being a manifestation of chronic diseases and inflammation. A low hemoglobin and hematocrit were associated with high mortality in PE in some research studies [10,15] and can increase the predictive ability of sPESI [11]. But anemia may be just a marker of comorbidity and chronic disease. An elevated MPV is correlated with platelet activation and adhesion. MPV correlates with right ventricular dysfunction (RVD) and was proved to predict PE recurrence and mortality in different research studies [7,8,16,17]. In contrast, other authors found no correlations between MPV and increased mortality, and this was explained by obtaining early complete blood counts, which may be prior to platelet changes and MPV increase [11]. An increased neutrophil level [6] or a decreased monocyte level were also significantly correlated with all-cause mortality in acute PE [18]. Several inflammatory cytokines like IL-8, monocyte chemoattractant protein-1 (MCP-1), interferon gamma (IFN-γ), epidermal growth factor (EGF), IL-6, tumoral necrosis factor alpha (TNF-α), vascular endothelial growth factor (VEGF), and IL-4 are elevated in PE, reflecting an increased systemic inflammatory response, and were also found to be predictors of PE severity [19]. Neutrophil-to-lymphocyte ratio (NLR) and platelet-to-lymphocyte ratio (PLR) are inflammatory biomarkers associated with endothelial dysfunction in patients with PE. NLR is known to be an independent prognostic factor for mortality in many cardiovascular and inflammatory diseases and is also studied in PE for the ability to identify high-risk PE patients. PLR and NLR were found to be correlated with all-cause mortality in PE and to improve the accuracy of sPESI for risk stratification in several studies [6,11,18,20,21]. In contrast, another study that investigated the role of hematological parameters, such as NLR, MPV, and WBC, in the prognosis of PE revealed no significant influence on early mortality. Furthermore, NLR, MPV, and WBC were significantly decreased after treatment [22].

In consequence, there are still many unresolved problems and incongruences regarding the prognostic value of blood cellular indices. Probably by using multiple cell counts, we can provide a more comprehensive evaluation of the associated inflammation. The development and resolution of thrombosis are associated with variations in the amount of inflammatory cells [23], and different timepoints in the evolution of PE are probably correlated with variability of hematological parameters [11].

Our research evaluated the value of different blood cellular indices as predictors of in-hospital adverse outcomes and mortality in patients with acute PE. The utility of the hematological parameters in conjunction with validated risk tools such as the PESI score and ESC mortality risk stratification was also studied. To our knowledge, the value of NLR in addition to the ESC risk stratification model was not evaluated before. Furthermore, the relationship between blood cellular indices and clinical status, respectively, PESI and sPESI scores, imagistic (echocardiographic or computed tomographic) indices of RVD, cardiac biomarkers (like high-sensitivity N-Terminal Pro-B-Type Natriuretic Peptide (NT-proBNP) and (hs) troponin), and ESC risk classes have been evaluated.

## 2. Materials and Methods

### 2.1. Study Design

This retrospective analysis included 1058 patients consecutively hospitalized in the Cardiology Department of Bihor County Emergency Clinical Hospital, Romania, between January 2019 and July 2023. Each case had a confirmed diagnosis of acute PE, verified through contrast-enhanced computed tomography pulmonary angiography (CTPA). Patient risk categorization was conducted in line with the ESC guidelines for PE severity assessment [2]. Exclusion criteria were as follows: patients with infections, chronic inflammatory conditions, known hematological diseases, ongoing cancer treatment, or immunosuppressant therapy. Patients with incomplete data were excluded as well. After applying these criteria, a total of 904 patients were included in the final analysis. Since this was a retrospective observational study, the sample size was not calculated *a priori* but was determined by the total number of eligible patients admitted during the study period. This approach allowed for a comprehensive evaluation of real-world clinical data and resulted in a robust sample size for statistical analysis. The number of patients was sufficient to support multivariate logistic regression, in line with the commonly accepted rule of at least 10 outcome events per variable. Given the observed adverse event rate of 18.25%, a post hoc power analysis confirmed that this sample size provided more than 80% power to detect clinically meaningful associations (e.g., odds ratios ≥ 1.5) at a conventional significance level of 0.05.

Blood was collected at admission with determination of complete blood count (CBC), NT-proBNP, and hs-troponin level. Transthoracic echocardiography was performed in the first 24–48 h after admission. Treatment was administered according to ESC guidelines. Most patients were treated with standard anticoagulation, either through intravenous unfractionated heparin or subcutaneous low-molecular-weight heparin. The decision to administer systemic thrombolysis with t-PA was made by the attending or on-call physician, depending on the patient’s hemodynamic condition.

In the hospital, adverse events were registered as follows: death or resuscitated cardiac arrest, occurrence of hemodynamic instability such as cardiogenic shock or the necessity of positive inotropic or vasoactive drugs, and systemic rescue thrombolysis.

Hematologic and other laboratory parameters and clinical and imagistic data were evaluated in patients with and without in-hospital adverse events, and those parameters that differed significantly were finally included in a multivariate logistic regression model.

This study was approved by the Ethics Committee of the Clinical County Emergency Hospital in Oradea, Bihor County, Romania (approval number 9411/08.04.2021) and conducted in compliance with the World Medical Association’s Declaration of Helsinki. Before being included in this study, each subject provided their informed consent.

### 2.2. Study Methodology

Confirmation of PE was achieved through CTPA by detecting an intravascular thrombotic obstruction within at least one segmental branch of the pulmonary artery. Based on clinical data, PESI and sPESI were evaluated for each patient at admission. The PESI risk score was calculated using an online tool [24]. Assessment relied on a composite of 11 clinical and physiological indicators, including patient age and sex, presence of cancer, history of cardiac failure or chronic respiratory illness, reduced arterial oxygen saturation under 90%, heart rate equal to or exceeding 110 beats per minute, low systolic blood pressure (SBP) below 100 mmHg, elevated respiratory frequency over 30 breaths per minute, neurological impairment, and temperature under 36 °C.

An alternative web-based calculator was employed to determine the simplified PESI (sPESI), which incorporates six clinical variables, namely, patient age, presence of neoplastic disease, chronic cardiac or pulmonary comorbidities, oxygen saturation in arterial blood falling under 90%, a heart rate of 110 beats per minute or higher, and SBP below 100 mmHg [25]. Imagistic (echocardiography and CTPA) data of RVD and elevated cardiac biomarkers were used for risk stratification of patients according to ESC guidelines [2].

Patients who presented with cardiac arrest or with clinical signs of shock (SBP < 90 mmHg or positive inotropic or vasoactive drug requirement and signs of hypoperfusion) or with persistent hypotension (SBP < 90 mmHg or a decrease in SBP ≥ 40 mmHg for more than 15 min, in the absence of other possible etiology such as a new-onset arrhythmia, hypovolemia, or sepsis) were included in the high-risk group. Patients with PESI class III–V or sPESI ≥ 1 were included in the intermediate-risk group. RVD on transthoracic echocardiography/CTPA and hs-troponin were also evaluated. Patients with both conditions, RVD and elevated hs-troponin, were in the intermediate high-risk group; if just one of the conditions was present, patients were assigned to the intermediate-low-risk group. Patients with a PESI class I–II or sPESI = 0, without RVD, were classified as low-risk patients [2].

Patient-related data were extracted from clinical documentation and the hospital’s electronic database. Demographic characteristics, medical antecedents, and potential risk contributors, along with clinical signs and investigative findings, were systematically documented. Upon admission, circulating cardiac biomarkers and complete blood counts were obtained. Quantification of NT-proBNP levels was performed using a chemiluminescent immunoassay system designed for cardiac marker detection, specifically the PathFast analyzer by LSI Medience Corporation (Tokyo, Japan). High-sensitivity troponin I concentrations were determined via the Alinity High Sensitive Troponin-I assay (Abbott Diagnostics, Illinois, USA). Hematologic indices were assessed using the CELL-DYN Ruby System (Abbott Laboratories, Illinois, USA), a fully automated analyzer capable of individual cell evaluation from a single dilution, employing Multi-Angle Polarized Scatter Separation (MAPSS) for leukocyte differentiation and laser-based optical scatter technology for erythrocyte and thrombocyte count.

Within the initial 24 to 48 h following hospital admission, echocardiographic assessments were conducted in patients diagnosed with PE, utilizing either the Siemens Acuson X300 system (Siemens Medical Solutions USA, Inc., Seoul, Korea) or the Philips CX50 POC platform (Philips Healthcare, Makati, Philippines), and the resulting cardiac imaging parameters were systematically documented. RVD was assessed by recording the following echocardiographic parameters: Right ventricle (RV) enlargement was evaluated by calculating the ratio between RV and left ventricle (LV) end-diastolic diameters measured at the level of mitral and tricuspid valve tips in apical four-chamber view. An RV/LV ratio greater than 1 was interpreted as indicative of right ventricular enlargement.

RV contractility was determined by measuring in M-mode the tricuspid plane annulus systolic excursion (TAPSE) that was considered diminished at <16 mm; hypokinesia of the RV free wall with good apical contractility (McConnel sign). Pulmonary artery systolic pressure (PASP) was estimated using the simplified Bernoulli equation, based on tricuspid regurgitation velocity obtained via Doppler imaging. The interventricular septum was evaluated visually for paradoxical motion or flattening. The diameter of the inferior vena cava was determined during end-expiration. LV ejection fraction was computed using the biplane Simpson’s method, applied to apical two- and four-chamber echocardiographic views.

CTPA scans were carried out using a 128-slice spiral CT (GE Medical Systems, LLC, 4855 West Electric Ave., Milwaukee, WI, USA). CTPA performed during the initial evaluation confirmed the presence of acute PE. RVD was evaluated by measuring the ratio between the largest RV and LV diameters in the axial plane 1 cm apical to the mitral and tricuspid valves. An RV/LV ratio > 1 was considered relevant for RVD.

PE-related death was defined as death that was confirmed at autopsy or appeared in a short time after a severe PE without other alternative diagnoses. Cardiac arrest was characterized by the requirement for initiation of cardiopulmonary resuscitation. Hemodynamic instability was characterized by the occurrence of cardiogenic shock with a persistent decrease in SBP < 90 mmHg for at least 15 min or the necessity of hemodynamic support, or the drop of SBP of >40 mmHg for more than 15 min, not explained by hypovolemia, sepsis, or arrhythmia. Systemic rescue thrombolysis refers to the necessity for therapy escalation prompted by the onset of hemodynamic instability during hospitalization.

### 2.3. Statistical Analysis

Statistical evaluation was performed using SPSS software version 25 [26] and MedCalc version 19.4 (MedCalc Software, Ostend, Belgium). Descriptive statistics were applied, with continuous variables summarized as means with standard deviations and categorical variables represented as absolute counts and percentages.

To assess differences between groups, continuous variables were compared using the independent samples *t*-test, while the chi-square test was selected for evaluating categorical data. A *p*-value below 0.05 was considered indicative of statistical significance.

Variables that showed significant differences in intergroup comparisons were further analyzed through multivariate logistic regression to determine their independent predictive value for adverse outcomes. A total of 16 variables met the criteria for inclusion in the regression model. To ensure the stability of the estimates, we followed the standard rule of at least 10 events per variable, which required a minimum of 160 adverse events. Given an observed adverse outcome rate of 18.25%, the available sample of 904 patients provided sufficient data for this model. A post hoc power analysis was performed using G*Power version 3.1 [27], assuming a two-tailed significance level of α = 0.05, an odds ratio of 1.5, and a base outcome probability of 18%. Under these conditions, this study achieved an estimated power of approximately 87%, confirming that the sample size was adequate to detect moderate effect sizes in the logistic regression analysis.

When comparisons involved more than two groups, one-way ANOVA was utilized for normally distributed variables, followed by the Tukey test to identify specific pairwise differences.

In cases where data did not meet parametric assumptions, the Kruskal–Wallis test was used, and the Bonferroni adjustment was applied to address the risk of Type I error due to multiple comparisons.

Correlation analyses (i.e., bivariate) were conducted to explore the relationships between hematological indices and key clinical markers such as PESI, sPESI, RVD, and relevant biomarkers including hs-troponin and NT-proBNP.

To evaluate the predictive capability of hematological markers for in-hospital adverse events, receiver operating characteristic (ROC) curve analysis was performed. The diagnostic accuracy of these parameters in forecasting mortality and complications was quantified by calculating the area under the ROC curve (AUC).

## 3. Results

**Figure 1:**
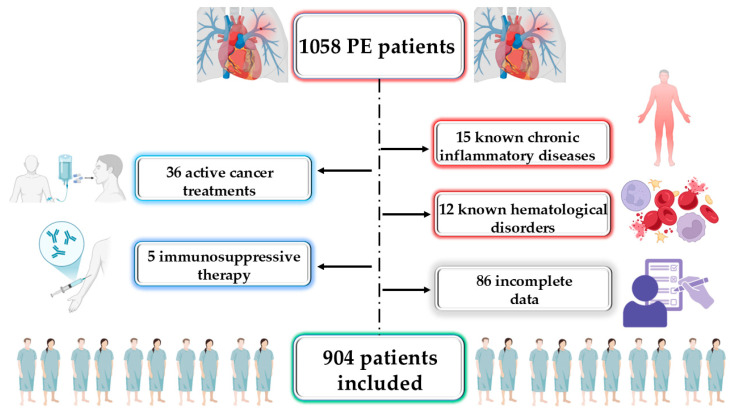
Study flow diagram. PE, pulmonary embolism.

A total of 1058 patients were consecutively admitted with a confirmed diagnosis of PE. Upon applying the exclusion criteria, 904 patients were eligible for inclusion in this study (Figure 1).

According to the classification provided by the ESC guidelines, 132 patients (14.6%) were categorized into the high-risk group, 567 patients (62.72%) were placed in the intermediate-risk category (i.e., comprising 217 in the intermediate-high and 350 in the intermediate-low subgroups), and 205 patients (22.67%) were assigned to the low-risk group. Following admission to the hospital, unfavorable outcomes, including in-hospital mortality, the emergence of cardiogenic shock or persistent hypotension, cardiac arrest, and the necessity for rescue thrombolysis, were observed in 165 patients (18.2%). Multiple events occurring in the same study participant were only counted once. A total of 131 patients (14.5%) experienced death related to PE. Moreover, 10 patients were resuscitated from cardiac arrest (1.1%) and were finally discharged alive. Hemodynamic instability and vasoactive or positive inotropic drug requirements were noted in 62 patients (6.9%). In the hospital, 22 patients suffered hemodynamic worsening that required escalation of therapy and rescue thrombolysis (2.4%).

### 3.1. Determinants of Negative In-Hospital Outcomes: Mortality, Need for Reperfusion Therapy, Hemodynamic Instability, and Resuscitated Cardiac Arrest

Baseline characteristics of the studied population are presented in Table 1. Patients that developed in-hospital adverse outcomes were older, more commonly smokers, and had more frequent previous histories of heart failure, coronary artery disease, diabetes, or stroke. SBP was significantly lower in patients with in-hospital adverse outcomes. Recent surgical intervention was more frequent in the adverse event group. As was expected, patients that developed in-hospital adverse events exhibited notably elevated levels of hs-troponin and NT-proBNP, more frequent RVD, and a higher PESI score.

Adverse outcomes were registered in most patients assigned after initial evaluation in the high-risk group, 121/132 (91.66%). In the intermediate risk group, 41 patients/567 (7.23%) (12/350 (3.42%) in the intermediate low and 29 from 217 (13.36%) in the intermediate high) had an adverse outcome during hospitalization. A number of 3/201 (2.14%) patients initially assigned to the low-risk group experienced an adverse outcome. Regarding hematological parameters, neutrophils were markedly increased in those who experienced adverse events, and NLR and PLR were substantially elevated in individuals experiencing in-hospital complications (Table 2).

In the context of multiple regression analysis, NLR remained an independent factor associated with in-hospital adverse events OR = 1.071 (95% CI 1.01–1.137), *p* = 0.02, together with PESI score OR = 1.042 (95% CI 1.025–1.059), *p* < 0.001, RVD OR = 4.389 (95% CI 1.162–16.580), *p* = 0.029, and hs-troponin OR = 1.002 (95% CI 1.002–1.003), *p* < 0.001.

**Figure 2:**
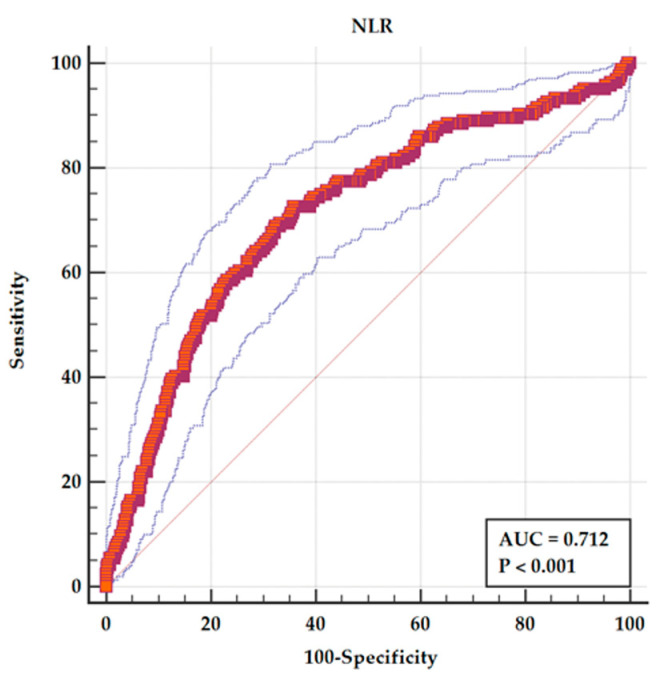
ROC analysis for NLR as a predictor of in-hospital adverse events. The solid red line represents the ROC curve for NLR, with an area under the curve (AUC) of 0.712 (*p* < 0.001), indicating fair discriminative ability. The dashed blue lines represent the 95% confidence interval (CI) for the ROC curve. The diagonal grey line indicates the line of no discrimination (AUC = 0.5).

The value of different hematological parameters as predictors of in-hospital complications was assessed by determining the AUC from ROC analysis and is revealed in Table 3. The highest value of AUC among hematological parameters as predictors of in-hospital complications was NLR (Figure 2), followed by neutrophils, lymphocytes, and leukocytes.

### 3.2. Distribution of Hematological Parameters According to the ESC Risk Classes

Hematological parameters were evaluated for every ESC risk group and are revealed in Table 4. NLR value increased with every ESC risk group, being significantly higher in the high-risk patients versus intermediate-risk and also in intermediate- versus low-risk patients. A notable parameter that demonstrated significant elevation in high-risk patients compared to those in the intermediate and low-risk categories was RDW.

Also, PLR, neutrophils, and leukocytes were significantly higher in high-risk patients compared to the other risk groups. Lymphocytes were significantly higher in the low-risk compared to the intermediate- and high-risk groups.

### 3.3. Correlation of Hematological Parameters with RVD, PESI Score, NT-proBNP, and Hs-Troponin

The following hematological parameters showed a direct, significant, but weak correlation in bivariate analysis with RVD: NLR had the highest correlation, r = 0.16 (95% CI 0.05–0.19), *p* = 0.001, followed by neutrophils, r = 0.15 (95% CI 0.03–0.17), *p* < 0.001, leukocytes, r = 0.13 (95% CI 0.04–0.15), *p* = 0.03, MPV, r = 0.11 (95% CI 0.03–0.17), *p* = 0.004, and RDW, r = 0.09 (95% CI 0.02–0.15), *p* = 0.007. An inverse weak correlation was present between RVD and lymphocyte r = −0.09 (95% CI −0.13–−0.02), *p* = 0.04. PLR, platelets, and Hb showed no correlation with RVD (*p* = NS).

A direct positive correlation was described with the PESI score for the following hematological parameters: NLR had the strongest correlation, r = 0.26 (95% CI 0.19–0.32), *p* < 0.001, followed by neutrophils, r = 0.17 (95% CI 0.11–0.24), *p* = 0.001, PLR, r = 0.17 (95% CI 0.11–0.23), *p* < 0.001, RDW, r = 0.15 (95% CI 0.09–0.22), *p* = 0.01, and leukocytes, r = 0.12, (95% CI 0.07–0.20), *p* = 0.03. An inverse correlation was observed with lymphocytes r = −0.13 (95% CI −0.19–−0.06), *p* < 0.001. There was no correlation between MPV and PESI score (*p* = NS).

The same direct significant correlation was found between sPESI and NLR r = 0.25 (95% CI 0.18–0.33), *p* < 0.001, RDW r = 0.16 (95% CI 0.10–0.22), *p* < 0.001, neutrophils r = 0.17, 95% CI 0.09–0.22), *p* < 0.001, and leukocytes r = 0.12, 95% CI 0.05–0.18), *p* = 0.005. There was a weak inverse correlation with lymphocyte r = −0.13 (95% CI −0.19–−0.07), *p* = 0.0001. MPV and PLR showed no correlation with sPESI (*p* = NS).

A direct weak correlation was observed between NT-proBNP and NLR r = 0.233, (95% CI 0.17–0.32), *p* = 0.004, RDW r = 0.228, (95% CI 0.16–0.31), *p* = 0.006, and MPV r = 0.218, (95% CI 0.16–0.32), *p* = 0.009. For the other hematological parameters, there was an absence of correlation with NT-proBNP. Troponin correlated with NLR r = 0.213 (95% CI 0.16–0.33), *p* = 0.032, but no significant correlation was found with the other hematological parameters.

### 3.4. Assessing the Influence of Demographics and Comorbidities on NLR Value as a Prognostic Tool in Pulmonary Embolism

The NLR was 4.87 ± 3.89 in patients aged <49 years, 6.40 ± 6.47 in those aged 50–75 years, and 8.23 ± 6.97 in patients > 75 years, with a statistically significant difference between age groups (*p* = 0.03). Women had a higher NLR (7.72 ± 7.57) compared to men (6.23 ± 5.08), *p* = 0.002.

Patients with a history of cardiovascular disease (CVD) had a mean NLR of 6.80 ± 5.54, compared to 7.23 ± 7.26 in those without a history of CVD (*p* = 0.32). Similarly, patients with a history of pulmonary disease had an NLR of 6.56 ± 5.83, versus 7.19 ± 6.78 in those without (*p* = 0.22). Diabetic patients showed a significantly higher NLR (7.98 ± 8.91) compared to non-diabetic patients (6.81 ± 5.80) (*p* = 0.03). In patients with hypertension, NLR was 6.65 ± 6.94, versus 7.41 ± 6.23 in those without hypertension (*p* = 0.08).

A linear regression was conducted to examine whether the interactions between NLR and different demographic and clinical characteristics of the study cohort predicted in-hospital outcomes in patients with pulmonary embolism.

No statistically significant interactions were observed between NLR and age (B = −0.006, t = −0.18, *p* = 0.85, 95% CI −0.04–0.03), sex (B = −0.17, t = −0.17, *p* = 0.86, 95% CI −0.06–0.05), smoking status (B = 0.21, t = 0.81, *p* = 0.35, 95% CI −0.30–0.72), history of CVD (B = −0.7, t = −1.81, *p* = 0.08, 95% CI −0.16–0.001), pulmonary disease (B = 0.05, t = 1.46, *p* = 0.14, 95% CI −0.002–0.01), hypertension (B = −0.04, t = −0.85, *p* = 0.39, 95% CI −0.01–0.004), or diabetes (B = −0.05, t = −1.25, *p* = 0.21, 95% CI −0.01–0.003).

### 3.5. Value of NLR in Combination with Validated Risk Scores—PESI and ESC Risk Scores

The ROC curve for sPESI as a predictor of adverse in-hospital outcome showed an AUC = 0.805 (95% CI 0.777–0.831), *p* < 0.001, for a cutoff value > 4 (95% CI > 3 > 4) with a sensitivity of 59.15% and a specificity of 89.84%.

**Figure 3:**
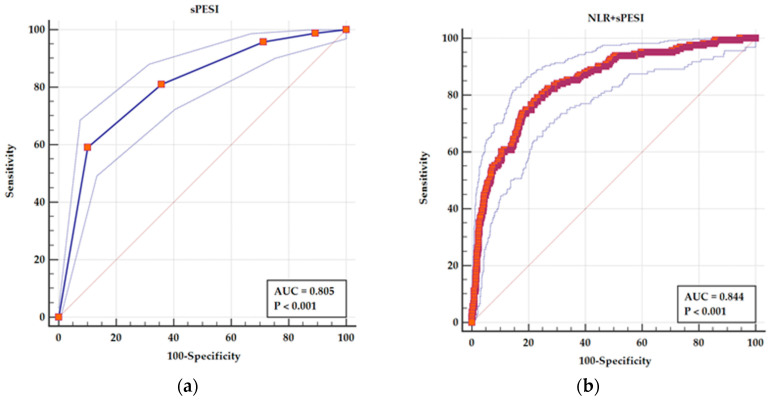
ROC curves for (**a**) sPESI as a predictor of adverse in-hospital events and (**b**) a combined parameters sPESI and NLR as a predictor of adverse in-hospital events. The solid line in each panel represents the ROC curve derived from the predictive model indicated. The outer dotted blue lines depict the 95% confidence interval (CI) for the area under the curve (AUC), reflecting the precision of the estimate. The diagonal line represents the line of no discrimination (AUC = 0.5). In (**a**), the blue ROC curve illustrates the predictive performance of sPESI alone (AUC = 0.805). In (**b**), the red ROC curve represents the combined model of sPESI and NLR, which shows an improved AUC of 0.844.

NLR had an acceptable AUC as a predictor of in-hospital adverse outcome. But in a combined model with sPESI, NLR is leading to an improvement in AUC from 0.805 (95% CI 0.777–0.831) for sPESI alone to AUC 0.844 (95% CI 0.819–0.868) for a combined parameter of sPESI and NLR (*p* < 0.001 for the difference between AUCs). This is leading to an improvement in sensitivity from 59.15% to 79.14%, with a specificity of 76.74% (Figure 3).

**Figure 4:**
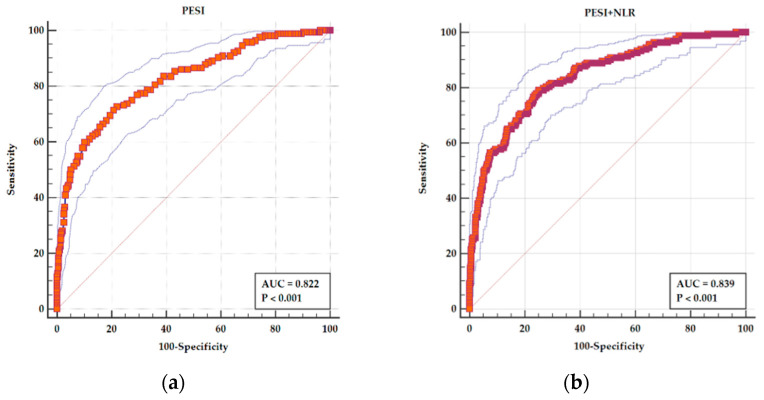
ROC curves for (**a**) PESI as a predictor of adverse in-hospital events and (**b**) a combined parameters PESI and NLR as a predictor of adverse in-hospital events. In both panels, the main ROC curve (solid stepped line) illustrates the relationship between sensitivity and 1-specificity for each model. The red diagonal line represents the line of no discrimination (AUC = 0.5), serving as a reference for random performance. The outer thin blue lines represent the 95% confidence interval (CI) bands, indicating the precision around the ROC estimate. The red squares mark the actual performance points used to construct the ROC curve. In panel (**a**), the PESI score alone demonstrates an AUC of 0.822, with sensitivity and specificity of 71.34% and 79.29%, respectively, at a cutoff >115. In panel (**b**), the combined model of PESI and NLR shows an improved AUC of 0.839, with higher sensitivity (79.14%) and slightly reduced specificity (74.83%), indicating a statistically significant improvement in predictive performance (*p* = 0.005).

By adding NLR, the AUC of PESI improved from 0.822 (95% CI 0.796–0.847), sensitivity of 71.34%, and specificity of 79.29% for a cutoff value of >115 to an AUC of 0.839 (95% CI 0.804–0.875) with an increase in sensitivity to 79.14% and a specificity of 74.83% (Figure 4). The difference between AUCs is statistically significant (*p* = 0.005).

From all the patients with adverse events, 121 (73.33%) were in the high-risk group and 29 (17.57%) were in the intermediate high-risk group. In our cohort, 12 patients (7.27%) with adverse events were in the intermediate-low group, and 3 patients (1.81%) with adverse events were in the low-risk class. From these patients with adverse outcomes, a number of 7 in the intermediate low-risk group and all the 3 patients in the low-risk group had an NLR ≥ 5.49. In consequence, by adding the NLR to ESC risk classification, an additional number of 10 (6.06%) patients from those who experienced complications could be reclassified in a higher risk group.

**Figure 5:**
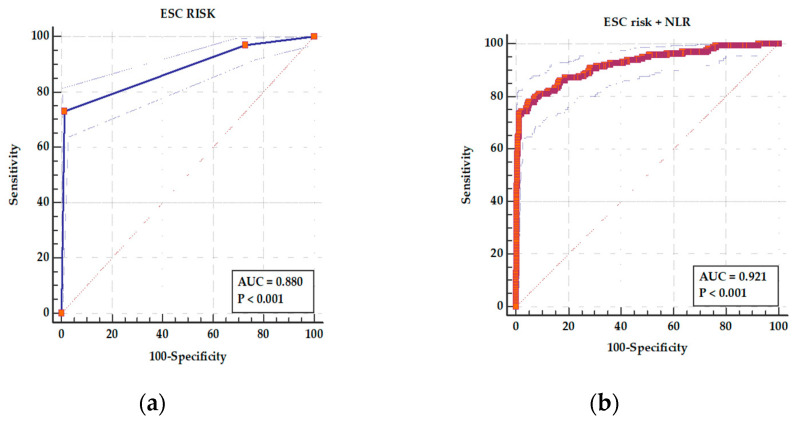
ROC curves for (**a**) ESC risk class as a predictor of adverse in-hospital events and (**b**) a combined parameters ESC risk class and NLR as a predictor of adverse in-hospital events. In panel (**a**), the ESC risk class yielded an AUC of 0.880 (95% CI: 0.857–0.901; *p* < 0.001), with a sensitivity of 73.17% and specificity of 98.64%. In panel (**b**), the combined ESC risk class and NLR model showed improved discrimination with an AUC of 0.921 (95% CI: 0.901–0.938; *p* < 0.001), sensitivity of 77.91%, and specificity of 94.77%. In both panels, the solid ROC curve represents the model performance, the surrounding dotted blue lines indicate the 95% confidence intervals, and the diagonal dashed line represents the line of no discrimination (AUC = 0.5). The improvement in predictive accuracy with the combined model was statistically significant (*p* < 0.001).

The ROC analysis showed an AUC of 0.880 (95% CI 0.857–0.901), *p* < 0.001, for ESC risk class as a predictor of in-hospital complications with a sensitivity of 73.17% and specificity of 98.64% (Figure 5a). For a combined parameter ESC risk class and NLR, the AUC increased to 0.921 (95% CI 0.901–0.938), *p* < 0.001, and sensitivity increased to 77.91% with a specificity of 94.77% (Figure 5b). The difference between areas was statistically significant (*p* < 0.001).

## 4. Discussion

The current study assessed the significance of hematological parameters in determining the risk levels of patients diagnosed with acute PE. The main finding of this research is that NLR can add to risk stratification of patients with acute PE. NLR was significantly elevated in patients who died or had in-hospital complications, and furthermore, the NLR persisted as an independent predictor of negative in-hospital outcomes, even after adjusting for confounding variables through multiple regression analysis. The value of NLR as a predictor of in-hospital complications is acceptable with an AUC of 0.712 determined by ROC curve analysis.

Thrombolysis is indicated in high-risk PE patients presenting with hypotension, cardiac arrest, or shock. However, hemodynamic instability or cardiac arrest may appear despite correct anticoagulant treatment in patients with initially normal blood pressure. Enhancing the accuracy of risk stratification in acute pulmonary embolism (PE) remains crucial to optimize the management of affected patients. PESI and its simplified version, sPESI, are established tools for evaluating the risk of early mortality, as outlined in the ESC guidelines [2]. These risk scores are composed of clinical parameters, and their sensitivity may be enhanced when utilized in connection with biomarkers of inflammation [22]. In the present study, NLR was found to increase the sensitivity and the predictive value of the PESI score and its simplified version.

Hematological parameter proportions have been assessed in numerous research studies regarding their diagnostic and prognostic performance in acute PE with variable results [24,25].

The correlation between elevated white blood cell (WBC) levels and acute PE was first described by Afzal et al. [28]. The WBC level was identified as an independent predictor of adverse events in hospitalized intermediate-risk acute PE patients in the study by Liu J. et al. [29]. A large study performed by Venetz et al. [10] on 14,228 patients with acute PE revealed an independent prognostic value of leukocyte count with a U-curve risk for 30-day mortality. Recently, in a multicenter, international registry, the Regional PE Registry (REPER), total WBCs were demonstrated to be markedly elevated in non-survivors with acute PE and improved risk stratification in intermediate–high-risk patients within the ESC risk classification [30]. Furthermore, leukocyte improved the risk stratification model for in-hospital all-cause death based on sPESI in a national, multicenter, prospective registry, the China Pulmonary Thromboembolism Registry Study (CURES) [29].

In our cohort, although leukocytes were increased in patients with adverse events, the difference between groups with and without an adverse outcome was not statistically significant.

Neutrophils and NLR levels are increased in patients with acute PE [31]. Neutrophils are initiating thrombus development, and platelets are involved in the propagation of venous thrombosis [32,33]. Neutrophils are entrapped in the developing thrombus and release neutrophil extracellular traps that can recruit other cells involved in the coagulation pathway [34]. An increased number of neutrophils can lead to increased VTE risk in patients with chronic heart, liver, or kidney disease [35], and the increased number of leukocytes in patients with myeloproliferative neoplasms increases the thrombotic risk [36]. In cancer patients, a higher incidence of VTE is linked to elevated NLR and PLR [36,37].

Furthermore, an increased neutrophil count was associated in several other studies with PE-related mortality, although in the study by Slajus B. et al., a correlation with mortality was not confirmed [6,18,21]. A decreased lymphocyte count was also associated with all-cause mortality [11]. NLR, a marker of subclinical inflammation, was proved to be an indicator of increased cardiovascular risk and mortality [38,39]. Additionally, research has demonstrated a significant correlation between NLR and 30-day mortality in PE, with NLR identified as an independent predictor of early mortality [40,41]. NLR was associated with massive embolism, PE being related to the thrombotic burden of PE [42].

Negative outcomes were associated with considerably higher neutrophil counts and NLR levels in our study sample.

The relationship between various hematological markers at the time of admission and the ESC risk stratification of acute PE was examined in our study. Our findings revealed that NLR escalated with each risk category defined by the ESC, with significantly higher levels in the high-risk group compared to the intermediate-risk group, and likewise in the intermediate-risk group compared to the low-risk group. Moreover, leukocyte and neutrophil counts were notably elevated in high-risk patients relative to those in the other risk categories. Conversely, lymphocyte levels were significantly more elevated in low-risk patients compared to those classified as intermediate or high risk. These observations align with the study by Peng et al., which identified a higher neutrophil count as an independent predictor of both intermediate- and high-risk PE [43].

The study performed by Kasapoğlu et al. also found significantly increased NLR in patients who died within 30 days of PE [44], but NLR was an independent predictor only in the subgroup of patients without comorbidities. But NLR proved to be an independent predictor of death in patients with acute PE in several other studies [33,43].

Ates et al.’s research demonstrated that NLR exhibited strong diagnostic accuracy in identifying massive PE (AUC = 0.893) [40]. NLR was a valuable biomarker that, added to the classical markers, such as cTnI and NT-proBNP, can predict RVD and 30-day mortality [45,46]. NLR was also significantly correlated with RVD in our study.

In the present study, the highest value among hematological parameters as predictors of in-hospital complications was NLR, followed by neutrophils, lymphocytes, and leukocytes. The same result was observed in recent published research performed by Yurtseven et al., where NLR was the strongest prognosticator, correlating with multiple clinical outcomes [45]. In addition, Ma et al.’s research corroborated the findings of this study, recognizing NLR as an independent factor for predicting 30-day mortality in acute PE, with an AUC of 0.792 (95% CI: 0.703–0.880, *p* < 0.001). For every unit of increase in NLR, the probability of death is increasing by 13.2% (OR = 1.13, 95% CI: 1.04–1.23) [21].

In our research, NLR as a discriminator between patients with and without hospital complications had an acceptable AUC. The sensitivity of NLR as a predictor of in-hospital adverse outcome was 72.56%, with a specificity of 64.19% for a cutoff value of 5.493 (95% CI 3.44–6.96). Furthermore, in multiple regression analysis, NLR remained an independent predictor of an adverse in-hospital outcome.

Ozcan S. et al.’s study discovered that NLR had a comparable cutoff value of 5.9, with a 68.4% sensitivity and 68.1% specificity for predicting in-hospital mortality [32].

NLR also significantly correlated with PESI and sPESI scores in our study and improved the value of these risk stratification tools by increasing their discriminative power and sensitivity as prognosticators of adverse outcomes. A strong correlation between PESI risk score and NLR, and an improvement in risk stratification by combining hematological parameters with PESI score, was also observed in other research studies [47,48].

Patients in the high-risk group and intermediate-high-risk group need close surveillance in the intensive care unit and more complex treatment (hemodynamic support, thrombolysis). But some of the patients that experienced adverse in-hospital outcomes remained undetected after the ESC risk classification was included after initial risk evaluation in intermediate-low or low-risk groups. There were 15 patients, 12 in the intermediate-low and 3 in the low-risk class, that experienced an adverse in-hospital outcome. From these patients, a number of 7 in the intermediate low-risk group and all 3 patients in the low-risk group had an NLR ≥ 5.49. In consequence, by adding the NLR to ESC risk classification, an additional number of 10 patients from those who experienced complications could be detected. This can lead to improved management and closer supervision of these patients. Furthermore, by combining ESC risk classification with NLR, an improvement in the AUC from 0.880 to 0.921 was observed with the increase in sensitivity for adverse event detection from 73.17% to 77.91%.

PLR was significantly higher in our study in high-risk patients versus intermediate- and low-risk patients. Although PLR was significantly elevated in patients who developed adverse in-hospital outcomes, the value of PLR as a predictor of in-hospital complications was poor, with a nonsignificant AUC, and in multiple regression analysis, it was not confirmed as an independent predictor of short-term complications.

The same results were obtained in the research by Ma Y. et al., where PLR was not confirmed as a predictor of short-term outcome in multivariate analysis. At the optimal cutoff value, the sensitivity of PLR was found to be comparatively low, given the severity of the condition, suggesting that PLR may not serve as an effective predictor for short-term mortality [21]. PLR was also evaluated as a diagnostic and prognostic marker in acute PE in several other studies. In cancer patients, PLR predicted the occurrence of VTE in the study by Grilz et al. [34], and an association was reported between PLR and the risk of post-surgery deep vein thrombosis [35]. Kurtipek et al. found elevated PLR in patients with acute PE versus healthy controls and suggested a correlation between PLR and pulmonary artery endothelial dysfunction [36]. Regarding the prognostic value of PLR, similar results to ours were obtained in another research on 550 patients with acute PE. Elevated PLR values were observed in patients who died within 30 days; however, PLR did not emerge as an independent predictor for 30-day mortality [42,49]. Any correlation was not observed between PLR and major adverse cardiopulmonary outcomes, although a weak association was described with in-hospital mortality (AUC = 0.610) [50] in the study performed by Ghaffari S. et al. on almost 500 patients with acute PE.

Different results were observed in several studies where PLR was found to be correlated with all-cause mortality [20,51,52,53] and with the PESI and sPESI scores. A prospective study on almost 500 patients with acute PE revealed that PLR was a significant independent predictor of in-hospital adverse events (OR: 1.588, 95% CI: 1.116–2.154, *p* = 0.004) [47]. The retrospective study by Trung Phan et al. on 191 patients revealed significantly higher values for PLR in high-risk patients compared to low-risk patients. The number of lymphocytes was decreased while there were minimal changes in neutrophils and platelets in this study [20], although in other research studies, a markedly elevated number of neutrophils associated with decreased lymphocytes was reported in non-survivors [6,21]. The variations are probably due to the variable inflammatory response reflecting the need for multiple markers to capture the immune response in PE. Lymphocyte decrease is correlated with the release of cortisol in the inflammatory context and of adrenaline during the sympathetic response [54].

A meta-analysis by Wang Q. et al., encompassing 7 studies with a total of 2323 patients, identified a correlation between NLR, PLR, and mortality. There was a significant correlation between NLR and short-term (in-hospital and 30 days) mortality (OR 8.43, 95% CI 5.23–13.61, *p* < 0.001) and between PLR and short-term (OR 6.69, 95% CI 2.86–15.66, *p* < 0.001) and long-term mortality (OR 6.11, 95% CI 3.90–9.55, *p* < 0.001) [49].

Multiple studies observed that PLR was increased in patients with massive PE with good diagnostic accuracy [40] and that PLR correlates with CT pulmonary obstruction index [47]. Furthermore, other studies observed increased PLR in patients with RV dysfunction [45,51,53]. PLR correlated with sPESI scores and was a distinct indicator of in-hospital death (AUC = 0.860), in the study by Kundi et al. [48]. In our investigation, a slight positive correlation was observed between PLR and PESI, whereas no association was noted with RVD.

An increased inflammatory status is associated with poor clinical outcomes in thromboembolic disease. Inflammation is a dynamic process, and serial measurements of NLR and PLR could perform better for prognostic assessment. Experimental models of PE revealed that neutrophils infiltrate the pulmonary arterial wall with a peak on day 2 and return to baseline on day 8, and macrophages have a peak on day 1 and return to baseline on day 4. Neutrophils infiltrated the RV wall between 6 and 18 h and resolved between day 4 and day 7, as was revealed by immune-histological analysis [55]. While inflammation worsens at the beginning of PE, there is an improvement during the evolution. Monitoring inflammation with PLR and NLR can help with following the response to therapy and the recovery during hospitalization.

But persistent inflammation may reflect an underlying disease such as infection, cancer, congestive heart failure, or chronic lung disease, and the elevation of PLR and NLR may be correlated with associated comorbidities. Inflammatory biomarkers are increased, and inflammation plays a complex role in the pathogenesis of PE. The endothelium is involved in the regulation of inflammation, vascular tone, and coagulation [51]. Several inflammatory biomarkers are demonstrated to increase in acute PE patients, like C-reactive protein; interleukins-6, 8, and 10; d-dimer; and matrix metalloproteinase-9 [56].

PE is a heterogeneous disease with a complex pathophysiology that involves activation of hemostasis, inflammation, cellular dysfunction, and hemodynamic impairment. Consequently, risk stratification and diagnosis remain challenging. The predictive value of various blood cellular indices for mortality is linked to their capacity to indicate the inflammatory process. Ratios between blood cellular indices seem to have the strongest correlation with inflammatory markers. In the study by Kantarcioglu et al., blood cellular indices ratios were elevated in patients with acute PE: NLR (8.22-fold), PLR (6.50-fold), and they systemic inflammatory index (SII) (5.52-fold), and they were able to predict 30-day mortality [56]. Also, in our study, the blood cellular ratio NLR was the best predictor of in-hospital complications among hematological parameters and persisted as an independent prognostic factor even after multiple regression analysis.

RBCs are increasing blood viscosity and pushing thrombocytes to the vessel wall, participating in thrombus generation [52]. The dimension variability of the erythrocyte is indicated by the red cell distribution width (RDW) [14,57] and is increased in VTE. Increased mortality associated with RDW has been observed in a range of cardiovascular diseases, such as pulmonary embolism, stroke, heart failure, peripheral artery disease, pulmonary arterial hypertension, and coronary artery disease [58,59].

The role of anisocytosis remains unclear, as it could either be a result, a contributing factor, or merely an incidental finding in various conditions linked to inflammation and heightened oxidative stress [20]. The Malmö Diet and Cancer study, conducted prospectively, revealed a dose-dependent, independent association between RDW and the likelihood of experiencing a first VTE event [14]. Patel GR’s study confirmed a stable and independent link between elevated RDW at various time intervals and the occurrence of the first DVT episode in adult patients [53]. In various studies, RDW has been identified as a reliable prognostic marker in patients with PE, serving as an independent predictor of early mortality related to PE [47,60]. The exact pathophysiological mechanism behind anisocytosis in PE is not fully understood, with RDW elevation being nonspecific and potentially reflecting chronic conditions and ongoing inflammation. But Ozcan et al. reported in a retrospective study that RDW is an independent risk factor for in-hospital mortality [32]. Furthermore, the predictive ability of RDW was superior to sPESI alone in the study by Slajus B. et al., and RDW supported the composite sPESI model becoming more accurate [11]. In Kasapoglu’s study, elevated RDW levels were observed in patients who succumbed within one month following the diagnosis of acute PE [44]. An increased level of RDW was useful as a prognostic factor for assessment of severity in PE patients in the study by Zhou et al. [61]. Also, risk stratification in PE was improved by combining RDW with PESI evaluation [62] in another research.

In our research, no statistically significant variations were found in RDW levels between patients with and without in-hospital complications. RDW failed to discriminate between patients with and without adverse in-hospital outcomes (AUC = 0.564) in the present study. But RDW was significantly increased in high-risk versus intermediate-risk and in intermediate- versus low-risk PE. RDW is probably a strong marker of increased mortality that reflects poor prognosis of different medical conditions. RDW is strongly correlated with conditions such as chronic inflammation, malnutrition, and chronic disease, and RDW may be just an unspecific chronic disease prognostic marker.

Circulating platelets are variable in size and activity. MPV is a measurement of platelet size and reflects platelet activation. Platelet consumption in the process of thrombosis will favor the release of large platelets from the bone marrow, and increased platelet activity is also stimulated by hypoxemia [16]. Elevated MPV was correlated with thrombosis and PE in several research studies [17,63] and MPV value was found to be elevated in patients who died, in contrast to those who survived [59,64], being a predictor of all-cause mortality [7,18]. The study performed by Hilal et al. [60] found no difference in both the intermediate- and high-risk acute PE groups’ MPV versus healthy subjects, but in non-survivors, MPV was higher as compared to survivors. There is also a recent meta-analysis that included 18 studies showing that MPV was significantly higher in patients with PE versus controls and in patients who died versus survivors [65].

In contrast, the study by Slajus B. et al. did not confirm the correlation between MPV and the mortality risk, and another research study performed by Kasapoglu et al., although it observed higher MPV levels in patients who died within 1 month after the diagnosis of acute PE, the difference was not statistically significant [11,44]. MPV had a significant direct correlation with RVD and with SBP in different studies. Furthermore, there was a significant relationship between MPV and modified Wells’ score, revised Geneva score, RV/LV ratio, pulmonary artery obstruction index, and length of hospital stay in the study by Abd El-Hady Abd El-Ghany et al. [66].

In this study, no noteworthy differences were observed concerning MPV values between patients who developed in-hospital adverse outcomes and those with favorable evolution. MPV levels were not different according to the ESC risk class. Although a weak correlation was found between MPV and RVD and between MPV and NT-pro BNP, MPV failed to predict the in-hospital prognosis of patients with acute PE in the present study.

There is a high variability of the results regarding the diagnostic and prognostic value of different blood cellular indices, which is explained by the various inflammatory milieus during thrombus development and resolution [23]. By determining blood cellular indices at different timepoints during the evolution of PE, variable results are obtained. Blood cellular indices are affordable and accessible and are often collected daily during hospitalization. It is worthwhile to assess complete blood count indices at multiple time points due to the evolving cellular and inflammatory nature of the thrombus [23]. A more comprehensive assessment of the inflammatory processes in PE can be obtained by combining multiple blood cellular indices. Furthermore, there are multiple differences between study populations enrolled in different research studies, and consequently blood cellular indices can reflect the presence of several chronic comorbidities and the poor prognosis associated with these chronic conditions. Future studies are required to assess the benefits of each blood cellular test and integrate them into a comprehensive risk model.

The value of the NLR as a prognostic marker in PE is supported by several criteria. First, there is biological plausibility: NLR is a biomarker that reflects systemic inflammation, and PE is a condition commonly associated with an inflammatory response. It is therefore plausible that a more intense inflammatory process would correlate with more severe PE and poorer outcomes. In our study, NLR levels increased in parallel with the severity of PE, suggesting its potential role in risk stratification and in identifying high-risk patients. This may contribute meaningfully to the clinical management of individuals with PE. Furthermore, NLR was found to correlate with established severity scores, such as the PESI and ESC risk classification scores.

Second, regarding the performance of NLR in predicting adverse outcomes, ROC curve analysis demonstrated acceptable discriminative ability, with moderate to good sensitivity and specificity. A cutoff value of 5.49 was identified as optimal for predicting adverse in-hospital outcomes. When NLR was added to well-validated risk scores such as the PESI and, especially, the ESC risk classification, a statistically significant improvement in the AUC (area under the curve) was observed. This combination resulted in a notable increase in both sensitivity and specificity, leading to improved detection of patients that will develop in-hospital complications and better management of these patients.

Third, to minimize confounding effects, patients with known inflammatory conditions, hematological disorders, active malignancies, or those undergoing immunosuppressive therapy, factors that could influence neutrophil or lymphocyte counts, were excluded from this study. Furthermore, NLR retained its significance in multivariate regression analysis as an independent predictor of in-hospital adverse events, even after controlling for confounding variables such as demographic characteristics, smoking status, comorbidities, other biological parameters, and echocardiographic parameters. It holds its value along with established clinical and biomarker indicators such as PESI, RVD, and hs-troponin, which are all recognized risk indicators, making it a clinically relevant and easily accessible tool for risk stratification. This supports the multifactorial nature of PE severity, where inflammation, hemodynamic condition, and myocardial injury all play key roles. The mean NLR was 7.05 ± 6.58 in our cohort of patients with PE, with values increasing progressively across ESC risk classes, from low to high risk. In contrast, NLR values in the general healthy population are significantly lower, typically ranging from 1.0 to 3.0, depending on age and other demographic factors [64].

Consequently, NLR is a widely available, easily measurable biomarker that can improve risk stratification in PE. However, it should be used as a complement to, rather than a replacement for, validated risk scores.

While NLR levels vary across demographic and clinical subgroups, particularly with age, sex, and diabetes status, no significant interactions were observed, indicating that NLR may serve as an independent prognostic marker in patients with PE across various demographic groups and clinical conditions.

This study has several limitations that should be considered when interpreting the results. Its retrospective and single-center design inherently carries the risk of selection bias and unmeasured confounding factors, which may influence the associations observed. Additionally, our analysis relied solely on hematological parameters collected at the time of admission. The absence of dynamic or serial measurements during hospitalization limits our understanding of how inflammatory markers might change over time and how such trends could relate to clinical outcomes. Future studies incorporating serial measurements would be valuable in capturing these dynamic trends. Another limitation is the narrow biomarker scope. This study focused exclusively on hematologic indices, without incorporating other potentially informative markers such as inflammatory cytokines or genetic factors. Including these might have provided further insight into the underlying biological mechanisms of PE and enhanced the precision of risk stratification. Moreover, this study examined only in-hospital outcomes, so we were not able to evaluate the prognostic utility of markers like the NLR beyond the acute phase. Long-term follow-up data, including events such as thromboembolism recurrence or the development of chronic thromboembolic pulmonary hypertension, would be essential to determine whether these markers retain their value over time and contribute to guiding extended patient management.

Nonetheless, the findings contribute to a better understanding of how routinely available hematological markers, such as NLR, may help support early clinical decision-making in patients with acute PE, even in the absence of more complex biomarker data.

## 5. Conclusions

NLR is an indicator of inflammation that integrates information from multiple cell lines and is the most valuable hematological biomarker for risk prediction and improved management in acute PE. NLR demonstrated an acceptable discriminative power regarding the identification of patients who experienced adverse in-hospital outcomes, enhancing both the sensitivity and predictive accuracy of the PESI score for complications and mortality. Furthermore, a combined model that is adding NLR to the ESC risk classification algorithm has improved accuracy and sensitivity for in-hospital adverse event detection.

## Figures and Tables

**Table 1:** Evaluation of baseline characteristics and paraclinical and clinical parameters in patients exhibiting and not exhibiting unfavorable in-hospital outcomes.

Variables	Total Cohort	Presence with Adverse Outcomes	Presence Without Adverse Outcomes	*p* Value
Adverse outcome	Total (904)	Yes (165)	No (739)	–
Age (years)	69.76 ± 14.379	73.08 ± 11.745	69.11 ± 14.775	0.002 ^1^*
Sex (F)	475/904 (52.54%)	83/165 (50.3%)	392/739 (53.04%)	0.26 ^2^
Smoking	215/904 (23.78%)	62/165 (37.57%)	153/739 (20.70%)	<0.001 ^2^*
History of cancer	131/904 (14.49%)	22/165 (13.33%)	109/739 (14.75%)	0.595 ^2^
History of CVD	678/904 (75%)	102/165 (61.81%)	576/739 (77.94%)	0.001 ^2^*
Heart failure	254/904 (28.097%)	54/165 (32.73%)	200/739 (26.67%)	<0.001 ^2^*
Coronary artery disease	174/904 (19.24%)	41/165 (24.74%)	133/739 (17.99%)	0.02 ^2^*
Valvular heart disease	202/904 (22.34%)	44/165 (26.66%)	158/739 (21.38%)	0.07 ^2^
Hypertension	425/904 (47.01%)	63/165 (38.18%)	362/739 (48.98%)	<0.001 ^2^*
History of pulmonary disease	200/904 (22.123%)	31/165 (18.79%)	169/739 (22.87)	0.659 ^2^
Diabetes	186/904 (20.57%)	46/165 (27.87%)	140/739 (18.94%)	<0.001 ^2^*
History of stroke	77/904 (8.51%)	23/165 (13.94%)	54/739 (7.31%)	<0.001 ^2^*
History of recent surgery	70/904 (7.74%)	9/165 (5.45%)	64/739 (8.66)	0.001 ^2^*
History of recent orthopedic surgery/major trauma	40/904 (4.42%)	6/165 (3.63%)	31/739 (4.19)	0.37 ^2^
Previous DVT/PE	109/904 (12.057%)	23/165 (13.84%)	86/739 (11.63%)	0.21 ^2^
BMI (kg/m^2^)	31.12 ± 7.07	33.62 ± 7.05	28.62 ± 7.8	0.61 ^1^
Systolic BP (mmHg)	125.88 ± 23.442	108.27 ± 29.847	128.89 ± 20.846	<0.001 ^1^*
HR	90.27 ± 18.85	98.57 ± 21.14	88.42 ± 18.34	0.32 ^1^
PESI score	103.659 ± 28.92	132.42 ± 31.36	97.04 ± 23.84	<0.001 ^1^*
sPESI	3.702 ± 1.023	4.34 ± 0.92	3.065 ± 1.126	<0.001 ^1^*
DVT	174/904 (19.24%)	13/165 (7.87%)	161/739 (21.78)	<0.001 ^2^*
Cholesterol (mg/dL)	229.73 ± 44.11	241.13 ± 33,21	218.33 ± 52.11	0.18 ^1^
NT-proBNP (pg/mL)	4714.84 ± 14,514.95	6851.866 ± 7715.463	3856.591 ± 16,478.369	0.03 ^1^*
Hs-Troponin (pg/mL)	250.464 ± 1143.271	1430.067 ± 2146.590	134.108 ± 403.271	<0.001 ^1^*
RVD (TTE/CTPA)	579/905 (63.97%)	129/165 (78.18%)	450/739 (60.89%)	<0.001 ^2^*
LVEF (%)	48.28 ± 7.80	47.91% ± 9.04	48.32% ± 7.64	0.71 ^1^

BP, blood pressure; BMI, body mass index; CTPA, computed tomographic pulmonary angiography; CVD, cardiovascular disease; DVT, deep vein thrombosis; Hs-Troponin, highly sensitive troponin; HR, heart rate; LVEF, left ventricular ejection fraction; NT-proBNP, N-terminal pro b-type natriuretic peptide; PE, pulmonary embolism; PESI, pulmonary embolism severity index; RVD, right ventricle dysfunction; TTE, transthoracic echocardiography; ^1^—*t*-test, ^2^—chi-square test, *—statistically significant (*p* < 0.05).

**Table 2:** Baseline hematologic parameters in patients with and without in-hospital adverse outcomes.

Variables	Total Cohort	Presence with Adverse Outcomes	Presence Without Adverse Outcomes	*p*-Value
Adverse outcome	Total (904)	Yes (165)	No (739)	–
Leukocytes (×10^3^/μL)	12.06 ± 5.48	13.98 ± 6.09	11.63 ± 5.24	0.116
Neutrophils (×10^3^/μL)	9.25 ± 4.77	11.43 ± 5.69	8.76 ± 4.40	0.02 *
Lymphocytes (×10^3^/μL)	1.88 ± 1.69	1.54 ± 1.16	1.96 ± 1.78	0.509
Platelets (×10^3^/μL)	227.635 ± 93.45	218.21 ± 100.88	229.73 ± 91.72	0.05
MPV (fl)	7.89 ± 1.669	7.88 ± 1.65	7.88 ± 1.65	0.074
Hb (g/dL)	13.56 ± 6.05	14.10 ± 13.49	13.45 ± 2.076	0.21
RDW (%)	13.22 ± 2.14	13.62 ± 2.42	13.12 ± 2.03	0.07
NLR	7.05 ± 6.58	10.88 ± 9.22	6.17 ± 5.46	<0.001 *
PLR	193.57 ± 129.254	366.93 ± 237.381	154.624 ± 114.857	<0.001 *

Hb, hemoglobin; MPV, mean platelet volume; NLR, neutrophils to lymphocyte ratio; PLR, platelet-to-lymphocyte ratio; RDW, red cell distribution width; *t*-test, * statistically significant (*p* < 0.05).

**Table 3:** ROC analysis of various hematological parameters as predictors of in-hospital adverse outcome.

CBC	AUC	*p*	Sensitivity %	Specificity %	Cutoff Youden Index
NLR	0.712 (95% CI 0.661–0.742)	<0.001 *	72.56	64.19	>5.493 (95% CI 3.44–6.96)
Neutrophils (×10^3^/μL)	0.668 (95% CI 0.636–0.699)	<0.001 *	75.61	56.67	>8.47 (95% CI 7.19–8.76)
Lymphocyte (×10^3^/μL)	0.657 (95% CI 0.624–0.688)	<0.001 *	64.63	64.97	<1.4 (95% CI 1.214–1.686)
Leukocyte (×10^3^/μL)	0.640 (95% CI 0.608–0.672)	<0.001 *	76.97	49.59	>10.63 (95% CI 9.8–11.3)
RDW (%)	0.564 (95% CI 0.53–10.597)	0.009 *	88.05	23.09	<11.65 (95% CI 11.27–13.04)
Hb (g/dL)	0.555 (95% CI 0.522–0.588)	0.033 *	61.96	51.86	<13.55 (95% CI <11.52–<15.96)
Platelets (×10^3^/μL)	0.548 (95% CI 0.514–0.581)	0.08	26.71	86.91	<148.2 (95% CI <134.6–<208.508)
PLR	0.534 (95% CI 0.500–0.567)	0.225	34.46	78.80	>190.58 (95% CI 45.36–288.93)
MPV (fl)	0.515 (95% CI −0.434–0.537)	0.577	28.3	78.8	<6.65 (95% CI 5.36–8.19)

AUC, area under curve; CBC, complete blood count; Hb, hemoglobin; MPV, mean platelet volume; NLR, neutrophils to lymphocyte ratio; PLR, platelet-to-lymphocyte ratio; RDW, red cell distribution width. ROC, curve analysis; * statistically significant (*p* < 0.05).

**Table 4:** Distribution of hematological parameters according to the ESC risk classes.

ESC-Risk Class	High Risk (132)	Intermediate Risk (567)	Low Risk (205)	*p*
Leukocytes (×10^3^/μL)	14.21 ± 6.56	11.84 ± 5.53	11.32 ± 4.05	<0.001 * (H vs. I and L)
Neutrophils (×10^3^/μL)	11.56 ± 6.14	9.06 ± 4.23	8.34 ± 4.77	<0.001 * (H vs. I and L)
Lymphocytes (×10^3^/μL)	1.62 ± 1.26	1.77 ± 1.25	2.39 ± 2.66	<0.001 * (L vs. I and H)
Platelets (×10^3^/μL)	220.873 ± 104.855	223.90 ± 89.94	243.067 ± 94.14	0.052
MPV (fl)	7.89 ± 1.85	7.91 ± 1.69	7.80 ± 1.60	0.884
Hb (g/dL)	14.19 ± 14.07	13.43 ± 2.09	13.52 ± 2.02	0.398
RDW (%)	13.92 ± 2.70	13.23 ± 1.97	12.77 ± 2.04	0.02 *
NLR	10.85 ± 9.68	7.07 ± 6.14	4.70 ± 3.87	<0.001 *
PLR	411.639 ± 2645.235	165.556 ± 126.385	134.322 ± 82.65	<0.005 * (H vs. I and L)

MPV, mean platelet volume; Hb, hemoglobin; RDW, red cell distribution width; NLR, neutrophils to lymphocyte ratio; PLR, platelet-to-lymphocyte ratio; H, high risk; I, intermediate risk; L, low risk. One-way ANOVA, * statistically significant (*p* < 0.05).

## Data Availability

The raw data supporting the conclusions of this article will be made available by the authors on request.

## Abbreviations

The following abbreviations are used in this manuscript:

RDW	Red Cell Distribution Width
RBC	Red Blood Cell
MPV	Mean Platelet Volume
VTE	Venous Thromboembolism
DVT	Deep Vein Thrombosis
AUC	Area Under the Curve
ESC	European Society of Cardiology
RVD	Right Ventricular Dysfunction
PESI	Pulmonary Embolism Severity Index
sPESI	Simplified Pulmonary Embolism Severity Index
WBC	White Blood Cell
NT-proBNP	N-Terminal Pro-B-Type Natriuretic Peptide
cTnI	Cardiac Troponin I
CI	Confidence Interval
OR	Odds Ratio
CT	Computed Tomography
RV	Right Ventricle
LV	Left Ventricle
SBP	Systolic Blood Pressure
PLR	Platelet-to-Lymphocyte Ratio
NLR	Neutrophil-to-Lymphocyte Ratio
CRP	C-Reactive Protein
PE	Pulmonary Embolism
ROC	Receiver Operating Curve
